# Epidemiological survey of human cytomegalovirus antibody levels in children from Southeastern China

**DOI:** 10.1186/1743-422X-11-123

**Published:** 2014-07-04

**Authors:** Qi Zhang, Yan Gao, Ying Peng, Miao Fu, Yan-Qing Liu, Qiu-Ju Zhou, Jian Yu, Xiao-Qun Zheng

**Affiliations:** 1 Department of Laboratory Medicine, The Second Affiliated Hospital & Yuying Children’s Hospital of Wenzhou Medical University, Wenzhou, Zhejiang, China; 2 School of Laboratory Medicine, Wenzhou Medical University, Wenzhou, China; 3 Key Laboratory of Laboratory Medicine, Ministry of Education, Wenzhou, China; 4 Department of Laboratory Medicine, Jinhua Municipal Central Hospital, Jinhua, Zhejiang, China; 5 Present adress: University of Missouri, Columbia, MO 65211, USA

**Keywords:** Human cytomegalovirus, Chemiluminescence immunoassay, Southeastern China, Child

## Abstract

**Background:**

This study investigated infection status and distribution of human cytomegalovirus (HCMV) serum markers in hospitalized children from the Wenzhou region.

**Methods:**

This survey was performed on 10,147 hospitalized children from birth to 14 years of age in Southeastern China (Wenzhou region) from March 2010 to March 2013. IgM and IgG antibodies to HCMV were quantitatively detected by chemiluminescence immunoassay (CLIA). HCMV IgM or IgG detection rates, concentration, and distribution in various age groups were retrospectively analyzed.

**Results:**

In this study of hospitalized children, the overall rates of HCMV IgM^+^ and IgG^+^ were 10.8% (1,099/10,147) and 83.0% (8,425/10,147), respectively. The lowest HCMV IgM^+^ rate (1.0%, *P* < 0.001) was observed in the group of patients <28 days of age whereas the highest HCMV IgM^+^ rate (19.9%, *P* < 0.001) occurred in the 28 days ~ 5 months old group. However, the concentrations of HCMV specific IgM in all age groups were not significantly different (*P* > 0.05). The HCMV IgG^+^ rate was highest in the <28 days group (98.1%, *P* < 0.001). The 28 days ~ 5 months old group had the lowest HCMV specific IgG concentrations (median, 133.9 AU/mL, *P* < 0.001). Among 1,099 HCMV IgM^+^ children, 405 (36.9%) were diagnosed with respiratory infections which pneumonia accounted for 18.2% (200/1,099) of the total population. However, children with respiratory infections had the lowest HCMV IgG concentrations (median, 161.1 AU/mL, *P* < 0.05).

**Conclusions:**

HCMV specific antibody responses are very common in hospitalized children with respiratory infection in Wenzhou region. Protection against HCMV airway infection needs greater emphasis and further studies will be helpful to reveal the role of HCMV in children respiratory disease.

## Introduction

The β-herpesvirus, human cytomegalovirus (HCMV), is globally ubiquitous in the general population [1,2]. Its prevalence varies from 60–80% to 80–100% in developed and developing countries, respectively, depending on geography, ethnicity, and socio-economic conditions [1,3]. Prevalence tends to be high in South America, Africa, and Asia, but low in Western Europe and the United States [4].

HCMV can be transmitted to the fetus during the entire pregnancy period, and is the most frequent cause of congenital infections during pregnancy [5,6]. More than 10–15% of congenitally infected newborns have symptoms at birth, and 5–15% of infected infants without symptoms will have adverse outcomes, including spontaneous abortion, neonatal death, or long-term neurologic sequelae (mental retardation, hearing loss, and visual impairment) [7-10]. Up to 60% of children are infected with HCMV in the first year of life with a wide range of clinical manifestations, such as pneumonia, infant hepatitis syndrome, and infectious mononucleosis. In older children, HCMV usually causes asymptomatic infections, although it may also be responsible for symptomatic glandular fever and hepatitis [11]. Following primary infection, the virus may remain latent and later reactivate to cause recurrent infection under certain situations such as reinfection with other viral strains [12,13].

However, data on HCMV infection prevalence in hospitalized children is scarce and most investigations have small sample sizes. Diagnosis of HCMV infection is mainly based on the HCMV specific IgG and IgM antibodies using enzyme-linked immunosorbent assay (ELISA) or chemiluminescence immunoassay in patient serum [14,15]. However, HCMV IgG and IgM couldn’t be correctly quantified by ELISA because of the poor reproducibility of the ELISA tests [16]. This study determined HCMV infection prevalence in hospitalized children from the Wenzhou region by detecting anti-HCMV IgG and IgM accurately using the AxSYM CMV IgM and IgG chemiluminescence immunoassay in order to monitor early HCMV infection and diagnosis in hospitalized children.

## Results

### HCMV seroprevalence

**Figure 1:**
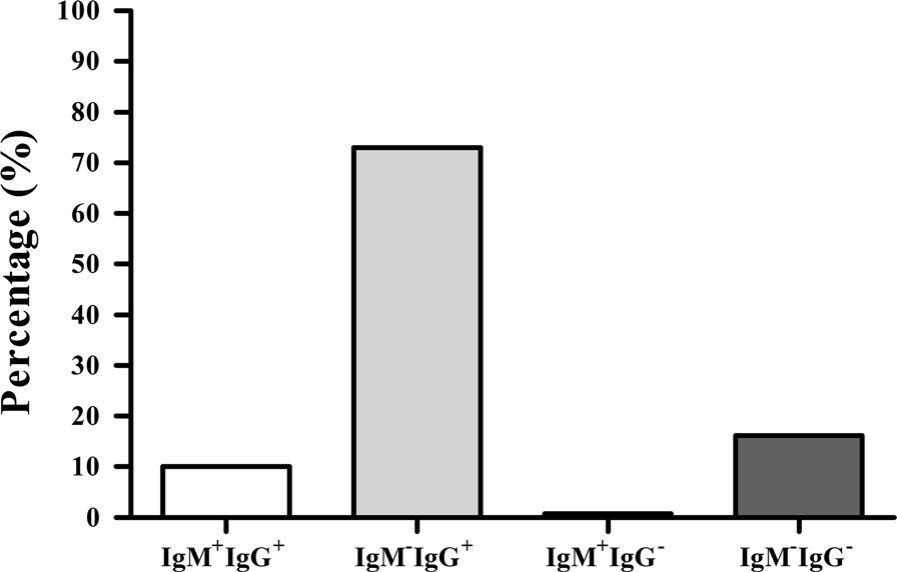
Serological results of HCMV antibodies in Wenzhou from 10,147 hospitalized children.

Of 10,147 hospitalized children, the overall rate of HCMV infection was 83.7% (8,509/10,147). The rates of overall IgM^+^ and overall IgG^+^ were 10.8% (1,099/10,147) and 83.0% (8,425/10,147), respectively. The rate of HCMV IgM^+^IgG^+^ was 10.0% (1,015/10,147), the rate of HCMV IgM^-^IgG^+^ was 73.0% (7,410/10,147), the rate of HCMV IgM^+^IgG^-^ was 0.8% (84/10,147), and the rate of HCMV IgM^-^IgG^-^ was 16.1% (1,638/10,147) (Figure 1, Table 1).

**Table 1:** Distribution of HCMV antibody results by age

**Age group**	**N**	**Overall IgM**^**+ **^**(%)**	**Overall IgG**^**+ **^**(%)**	**IgM**^**+ **^**IgG**^**- **^**(%)**	**IgM**^**+ **^**IgG**^**+ **^**(%)**	**IgM**^**- **^**IgG**^**+ **^**(%)**	**IgM**^**- **^**IgG**^**- **^**(%)**
<28 days	1,137	11 (1.0)	1,114 (98.1)	1 (0.1)	10 (0.9)	1,104 (97.2)	22 (1.9)
28 days	3,205	639 (19.9^a^)	3,074 (95.9^c^)	3 (0.1)	636 (19.8)	2,438 (76.1)	128 (4.0)
6 months	1,828	115 (6.3^a b^)	1,342 (73.4^c^)	16 (0.9)	99 (5.4)	1,243 (68.0)	470 (25.7)
1 year	1,755	168 (9.6^a b^)	1,124 (64.1^c e^)	36 (2.1)	132 (7.5)	992 (56.5)	595 (33.9)
3 years	1,424	128 (9.0^a b^)	1,079 (75.8^c e^)	25 (1.8)	103 (7.2)	976 (68.5)	320 (22.5)
7 ~ 14 years	798	38 (4.8^a b^)	692 (86.7^c^)	3 (0.4)	35 (4.4)	657 (82.3)	103 (12.9)
Total	10,147	1,099 (10.8)	8,425 (83.0)	84 (0.8)	1,015 (10.0)	7,410 (73.0)	1,638 (16.1)

Chi-square test.

^a^*P* < 0.001 compared to <28 days.

^b^*P* < 0.001 compared to 28 days.

^c^*P* < 0.001 compared to <28 days.

^e^*P* < 0.001 compared to 7 ~ 14 years.

### Detection of HCMV antibody in different age groups

Differences in HCMV antibody levels among the 6 age groups were observed. (1) The <28 days group had the lowest HCMV IgM^+^ rate (1.0%), which was significantly different from the values for the other age groups (*P* < 0.001). (2) The HCMV IgM^+^ rate (19.9%) in the 28 days group was distinctly higher than the other groups (*P* < 0.001). (3) The highest HCMV IgG^+^ rate (98.1%), observed in the <28 days group, was significantly different from the other groups (*P* < 0.001). (4) Infants younger than 12 months were excluded due to the potential for maternal HCMV-specific IgG [17,18]. Therefore, only groups aged 1–2 years, 3–6 years, and 7–14 years were compared. The 7–14 years group had the highest HCMV IgG^+^ rate (86.7%) among these three groups, a statistically significant difference (*P* < 0.001) (Table 1).

### HCMV IgG concentration in seropositive individuals according to age

There were no significant differences in HCMV IgM antibody concentration among the <28 days, 28 days-5 months, 6–12 months, 1–2 years, 3–6 years, and 7–14 years groups (*P* > 0.05). A significant difference in HCMV IgG concentration was observed among these 6 groups (*P* < 0.01). The 28 days-5 months group (median: 133.9 AU/mL) had a significantly lower HCMV IgG level than the other groups (*P* < 0.001) (Table 2).

**Table 2:** HCMV IgG concentration in seropositive individuals by age group

**Age**	**HCMV-IgG positive**	**IgG concentration (median, AU/mL)**	***P***
<28 days	1,114	248.1	<0.01^*^
28 days^△^	3,074	133.9	<0.01^*^
6 months	1,342	250	<0.01^*^
1 year	1,124	250	<0.01^*^
3 years	1,079	194.2	<0.01^*^
7 ~ 14 years	692	191.9	<0.01^*^

Note: ^△^: 28-day group is significantly different from all other groups (*P* < 0.001, Wilcoxon rank sum test).

*: there is a significant difference in HCMV IgG concentration distribution of these 6 groups (*P* < 0.01, Kruskal-Wallis H test).

### Disease distribution in HCMV IgM^+^ individuals

The 1,099 HCMV IgM^+^ individuals had respiratory infections (36.9%), hepatobiliary disease (17.7%), and blood disorders (16.5%) (Table 3). Viral DNA was detected using real-time polymerase chain reaction (RT-PCR) of bronchoalveolar lavage (BAL) fluid from 58 HCMV IgM^+^ with respiratory infections, of which 47 were positive by both IgM detection and RT-PCR whereas 11 cases were only IgM-positive (Table 4).

**Table 3:** Distribution of diseases in individuals positive for HCMV IgM

**Related diseases**	**Age (median, in months)**	**N (%)**
*Respiratory infections*	3 (0–144)	405 (36.9)
Pneumonia	2 (0–120)	200 (18.2)
Bronchiolitis	3 (1–24)	65 (5.9)
Upper respiratory tract infection	10 (1–144)	52 (4.8)
Bronchopneumonia	3 (1–48)	43 (3.9)
*Blood disorders*	24 (0–168)	181 (16.5)
Thrombocytopenia	3 (0–120)	69 (6.3)
Infectious mononucleosis	48 (4–168)	68 (6.2)
*Hepatobiliary disease*	2 (0–144)	194 (17.7)
Infant hepatitis syndrome	2 (1–12)	95 (8.6)
Jaundice	2 (0–120)	33 (3.0)
*Gastrointestinal diseases*	3 (1–48)	79 (7.2)
Diarrhea	3 (1–12)	41 (3.7)
Enteritis	5 (1–24)	20 (1.8)
*Infectious disease*	6 (1–144)	75 (6.8)
Septicemia	2 (1–36)	29 (2.6)
*Nervous system disease*	11 (0–72)	27 (2.5)
Convulsions	8.5 (1–72)	18 (1.6)
*Urinary system disease*	3 (1–132)	21 (1.9)
Urinary tract infection	2 (1–12)	15 (1.4)
*Fever of undetermined origin*	24 (2–72)	25 (2.3)
*Other non-specific diseases*	4 (0–168)	92 (8.4)
Total	4 (0–168)	1,099 (100)

**Table 4:** **PCR detection of BAL HCMV DNA from 58 HCMV IgM**^**+ **^**individuals with different respiratory infections**

**Respiratory infections subcategories**	**N**	**Mean age (range months)**	**PCR detection results (N)**	
			**Positive**	**Negative**
Pneumonia	35	3.2 (1-24)	28	7
Bronchiolitis	22	3.2 (1-9)	19	3
Upper respiratory tract infection	1	24	0	1
Total	58	3.6 (1-24)	47	11

### Analysis of HCMV IgG concentration in HCMV IgG^+^ individuals according to disease

**Figure 2:**
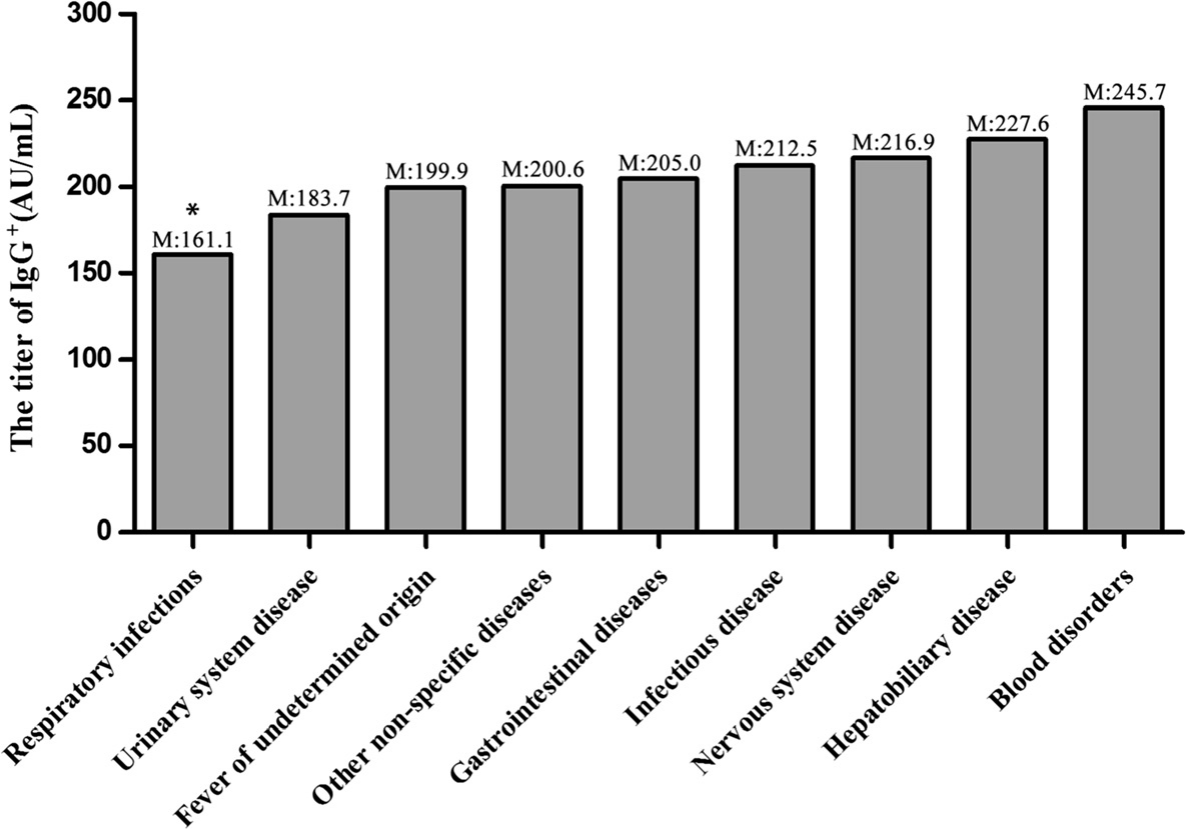
**HCMV IgG concentration in HCMV IgG**^**+ **^**individuals with system diseases.** The group of patients with respiratory infections has the lowest HCMV IgG concentration, a statistically significant difference compared to the other groups (median, 161.1 AU/mL, *P* < 0.05, Wilcoxon rank sum test). M represents the median HCMV IgG concentration within each group.

HCMV IgG concentration was significantly lower in individuals with respiratory infections compared to other groups (median, 161.1 AU/mL, *P* < 0.05). No significant differences were observed between the group with blood disorders, which had the highest HCMV IgG antibody concentration (median, 245.7 AU/mL, *P* > 0.05), and other groups (Figure 2).

## Discussion

In general, HCMV is acquired earlier in life among lower socioeconomic strata in developed countries and in developing countries [19]. In some African nations, HCMV seroprevalence is as high as 80–90% by 10 years of age; in contrast, seroprevalence is below 20% by 15 years of age among subgroups of children in the United States and Great Britain. Several studies have reported HCMV seropositivity rates in some parts of China. One study showed a more than 70% overall rate of HCMV infection among the children younger than 15 years old in the Shanghai area (Southeastern China) [20]. In Eastern China, several studies showed lower HCMV seroprevalence. Zhao et al. reported a 33.5% HCMV seroprevalence in Jinan (Eastern China) among people younger than 20 years of age [21] and Sun et al. showed a 42.5% HCMV seroprevalence in people younger than 6 years old in the Weifang area (Eastern China) [22]. Our data show a 83.7% HCMV infection rate among hospitalized children in Wenzhou, higher than the study by Fang et al. , significantly higher than in Eastern China, indicating that HCMV is an important pathogen in hospitalized children of Southeastern China. The reasons for this high infection rate may be related to geography, socio-economic conditions, or sensitivity differences between testing methodologies.

Our data show that children less than 28 days of age had the highest HCMV IgG^+^ and lowest HCMV IgM^+^ rates (Table 1). In addition, statistical analysis showed the 28 days group to have the highest HCMV IgM^+^ rate and the lowest HCMV IgG antibody concentrations. The reasons for these observations may include: (i) postnatal transmission of HCMV through breast milk [23-25]. HCMV is reactivated in latently infected mothers during lactation and breast-fed infants are susceptible to HCMV infection from breast milk, so infants are at risk for primary HCMV infection [26,27]. Because the infant cell-mediated and humoral immune system is immature, some infants (particularly <28 days of age) cannot produce an effective response to HCMV infections. (ii) The half-life of specific maternal IgG in infants is about 20 to 30 days [28,29]. As infants are exposed to external HCMV, maternal HCMV-specific IgG may be depleted to clear the virus. The HCMV IgG concentration in the 28 days age group was much lower than the high concentration observed in the <28 days age group, suggesting that the 28 days group may have increased risk of active HCMV infection (Table 2).

HCMV has a diverse tropism in its host and infects most types of cells, including epithelial and endothelial cells, fibroblasts, smooth muscle cells, and various hematopoietic cells [30]. During congenital infection, fetal lungs are particularly targeted by HCMV [31,32], leaving patients vulnerable to respiratory diseases, which account for 36.9% of all HCMV cases (Table 3). HCMV IgM^+^ individuals with respiratory infections showed that 11 of 58 cases were negative by Real-time PCR assay. The reason for this result may be that HCMV IgM was likely present, but the infection had been resolved. The liver is one of the most vulnerable organs infected by HCMV [33], and HCMV is the leading cause of infant hepatitis syndrome (IHS) [34]. In this study, IHS accounts for 8.6% in HCMV IgM^+^ patients. Although serological results suggest HCMV infection, further study is needed to distinguish whether HCMV infection and/or mixed infections with other pathogens results in these diseases.

## Conclusions

In summary, this is the first study to our knowledge to screen for HCMV infection in hospitalized children in Wenzhou (Southeastern China). We observed a high HCMV prevalence rate of 83.7%. Although no evidence was shown that HCMV was the cause of these respiratory infections, respiratory infection is a leading disease among hospitalized children positive for HCMV antibodies. The high prevalence of HCMV infection emphasizes HCMV diagnosis, prevention, and therapy for young children.

## Methods

### Ethics statement

This study was approved by the Ethics Committee of Wenzhou Medical University. Written informed consent was obtained for all study participants.

### Serum samples

The serum samples used in this study were obtained from 10,147 children hospitalized at the Second Affiliated Hospital of Wenzhou Medical University between March 2010 and March 2013. The hospitalized children were classified into 6 sub-groups according age: 1–27 days, 28 days-5 months, 6–12 months, 1–2 years, 3–6 years, and 7–14 years.

### Bronchoalveolar lavage fluid samples for PCR detection of HCMV DNA

Bronchoalveolar lavage (BAL) samples from 58 HCMV IgM^+^ individuals with respiratory infections (see Table 4 for detail information) were collected from pediatric patients with respiratory disease during their initial visits to their doctors. These 58 hospitalized patients were chosen for PCR detection of HCMV DNA from BAL because of their HCMV IgM positivity and clinical symptoms associated with HCMV infection. BAL were sampled using standard techniques, and the specimens were stored at -80°C until RT-PCR was performed.

### Serology

Patient blood samples were collected from the department of internal medicine. Two milliliters of whole blood from each patient were obtained and stored at 4°C for 4 hours; serum was then separated by centrifugation at 4,000 × rpm for 10 min at 4°C. Sera were frozen at -20°C before measuring HCMV antibody concentrations. HCMV IgM and IgG were detected using a commercial microparticle chemiluminescence immunoassay (LIA) (AxSYM, Abbott Laboratories, USA) according to the manufacturer’s instructions. Based on manufacturer’s recommendations, HCMV-specific IgM index values ≥0.5 and HCMV-specific IgG values >15.00 AU/mL are considered positive, and the IgG upper limit is 250.00 AU/mL.

### Quantitative PCR for HCMV DNA

Nucleic acids (50 μL) were extracted from 1 ml BAL specimens. Two μL of the extracted DNA was subjected to each RT-PCR reaction using a commercially available Diagnostic kit for Quantification of Human Cytomegalovirus DNA (DAAN, China) following the manufacturer’s instructions. RT-PCR assays were performed using the 7500 Real Time PCR System (Applied Biosystems, USA). HCMV DNA levels were reported as number of DNA copies per ml bronchoalveolar lavage fluid. The limit of detection of this quantitative assay is 500 copies/mL. The PCR condition is: initial denaturation step at 93°C for 2 min, 10 cycles of denaturing at 93°C for 45 s and annealing and extension at 55°C for 1 min, 30 cycles of denaturing at 93°C for 30 s and annealing and extension at 55°C for 45 s.

### Statistical analysis

The laboratory data were built into a Microsoft Excel database. Chi-squared, Kruskal-Wallis H, and Wilcoxon rank sum tests were performed to detect significant correlations between groups using SPSS Statistics for Windows, version 17.0 (SPSS, Inc., Chicago, IL, USA). *P* < 0.05 was considered statistically significant.

## Abbreviations

HCMV: Human cytomegalovirus; CLIA: Chemiluminescence immunoassay; BAL: Bronchoalveolar lavage; RT-PCR: Real-time polymerase chain reaction.

## Competing interests

The authors declare that they have no competing interests.

## Authors’ contributions

QZ, YG, YP and X-QZ conceived and designed the experiments. QZ, YG, and MF contributed to data collection and performed the experiments. QZ, Q-JZ, and YP participated in data and statistical analyses. QZ wrote the manuscript. Y-QL, JY, and YP revised the manuscript. All authors read and approved the final manuscript.
